# In vitro activity of exebacase against methicillin-resistant *Staphylococcus aureus* biofilms on orthopedic Kirschner wires

**DOI:** 10.1186/s13104-023-06468-y

**Published:** 2023-09-11

**Authors:** Melissa J. Karau, Jay Mandrekar, Dario Lehoux, Raymond Schuch, Cara Cassino, Robin Patel

**Affiliations:** 1 Division of Clinical Microbiology, Department of Laboratory Medicine and Pathology, Mayo Clinic, 200 First Street SW, Rochester, MN 55905 USA; 2 Division of Biomedical Statistics and Informatics, Department of Health Sciences Research, Mayo Clinic, Rochester, MN 55905 USA; 3 ContraFect Corporation, Yonkers, NY 10701 USA; 4 Stony Point Life Sciences Consulting, LLC, Benson, VT 05743 USA; 5 Division of Public Health, Infectious Diseases, and Occupational Medicine, Department of Medicine, Mayo Clinic, 200 First Street SW, Rochester, MN 55905 USA

**Keywords:** MRSA, Lysins, Exebacase, Biofilms, *Staphylococcus*

## Abstract

Orthopedic foreign body-associated infection can be difficult to treat due to the formation of biofilms protecting microorganisms from both antimicrobials and the immune system. Exebacase is an antistaphylococcal lysin (cell wall hydrolase) under consideration for local treatment for biofilm-based infections caused by methicillin-resistant *Staphylococcus aureus* (MRSA). To determine the activity of exebacase, we formed MRSA biofilms on orthopedic Kirschner wires and exposed them to varying concentrations (0.098, 0.98, 9.8 mg/ml) of exebacase and/or daptomycin over 24 h. The biofilm consisted of 5.49 log_10_ colony forming units (cfu)/K-wire prior to treatment and remained steady throughout the experiment. Exebacase showed significant biofilm reduction at all timepoints (up to 5.78 log_10_ cfu/K-wire; P < 0.0495) compared to the controls at all concentrations and all time points with bactericidal activity (> 3 log_10_ cfu/K-wire reduction) observed for up to 12 h for the 0.098 and 0.98 mg/ml concentrations and at 24 h for 9.8 mg/ml. Daptomycin showed significant biofilm reduction, although non-bactericidal, at all time points for 0.98 and 9.8 mg/ml and at 4 and 8 h with 0.098 mg/ml (P < 0.0495). This study supports further evaluation of local administration of exebacase as a potential treatment for orthopedic implant infections.

## Introduction

Orthopedic implant related infections represent a challenging issue in patients with implanted instrumentation or medical devices. The location of the orthopedic implant offers bacteria an environment to evade both systemic antibiotics and the immune system. When implants become infected, bacteria often form protective biofilms, and colonize the local bone tissue by invading the osteocyte lacuno-canalicular network and entering cells such as osteoblasts and fibroblasts, shielding themselves from neighboring immune cells [1, 2]. These areas also have low blood supply, limiting access of antibiotics delivered systemically. Surgery is often needed to debride infected sites, sometimes with implant removal [3]. Local treatment options are limited but would be beneficial in treating these hard-to-reach infections.

Exebacase is a recombinantly produced peptidoglycan hydrolytic enzyme that elicits rapid cell wall destabilization and concomitant osmotic lysis of staphylococci. Exebacase exhibits (i) rapid, targeted bactericidal activity; (ii) synergy with antistaphylococcal antibiotics, including daptomycin (DAP) and vancomycin; (iii) a low propensity for the development of resistance; (iv) no cross-resistance with antibiotics; (v) the capacity to both suppress antibiotic resistance and “re-sensitize” antibiotic-resistant bacteria; and (vi) in vitro and in vivo postantibiotic effects [4–13]. In vivo activity has been demonstrated in experimental animal models of *S. aureus* infection, including murine bacteremia [8], pneumonia [10] and thigh infection [14]; rat osteomyelitis [15]; and rabbit endocarditis [16] and implant related infection [17]. Here, exebacase (ContraFect, Yonkers, NY) and/or daptomycin (Teva Pharmaceuticals USA Inc., North Wales, PA) at varying concentrations were tested against methicillin-resistant *Staphylococcus aureus* (MRSA) biofilms on orthopedic Kirschner wires (K-wires).

## Methods

Biofilms were established on 5 × 1.1 mm threaded stainless steel K-wires (Zimmer Biomet, Warsaw, IN) in 1 ml containing 10^6^ cfu/ml of MRSA IDRL-6169 (clinical MRSA isolate from a periprosthetic hip infection at Mayo Clinic) in Bacto™ Tryptic Soy Broth (BD, Sparks, MD) (TSB) at 37 °C on an orbital shaker. After 10 h, K-wires were randomly transferred to one of nine treatment groups in triplicate. The two control groups were the exebacase carrier (20 mM L-histidine and 5% D-sorbitol) and the daptomycin carrier (saline). Exebacase and daptomycin each were tested at 0.098, 0.98 and 9.8 mg/ml. The combination of exebacase and daptomycin at 0.098 mg/ml each was also tested. K-wires were placed into 40 μl of solution and treated for 2, 4, 8, 12 or 24 h, after which they were removed from the treatment solution and rinsed in sterile saline to remove remnant solution that could impede organism recovery. To recover any remaining viable biofilm, K-wires were placed into 0.5 ml of sterile saline, vortexed for 30 s, sonicated for 5 min (40 kHz, 0.22 W/cm^2^) and vortexed an additional 30 s. Sonicate fluid was serially diluted and plated onto Trypticase™ Soy Agar with 5% Sheep Blood (BD, Sparks, MD) and 3 ml TSB was added to the remaining sonicate fluid. Cultures were incubated for 48 h at 37 °C. Results were reported as mean log_10_ colony forming units (cfu)/K-wire. Positive broth cultures were reported as 0.65 log_10_ cfu/K-wire and the limit of detection was 0.13 log_10_ cfu/K-wire. Statistical analyses were performed using SAS software version 9.4 (SAS Inc, Cary, NC) with the Wilcoxon rank sum test for making pairwise comparisons between the groups. P-values less than 0.05 were considered significant.

## Results

**Fig. 1:**
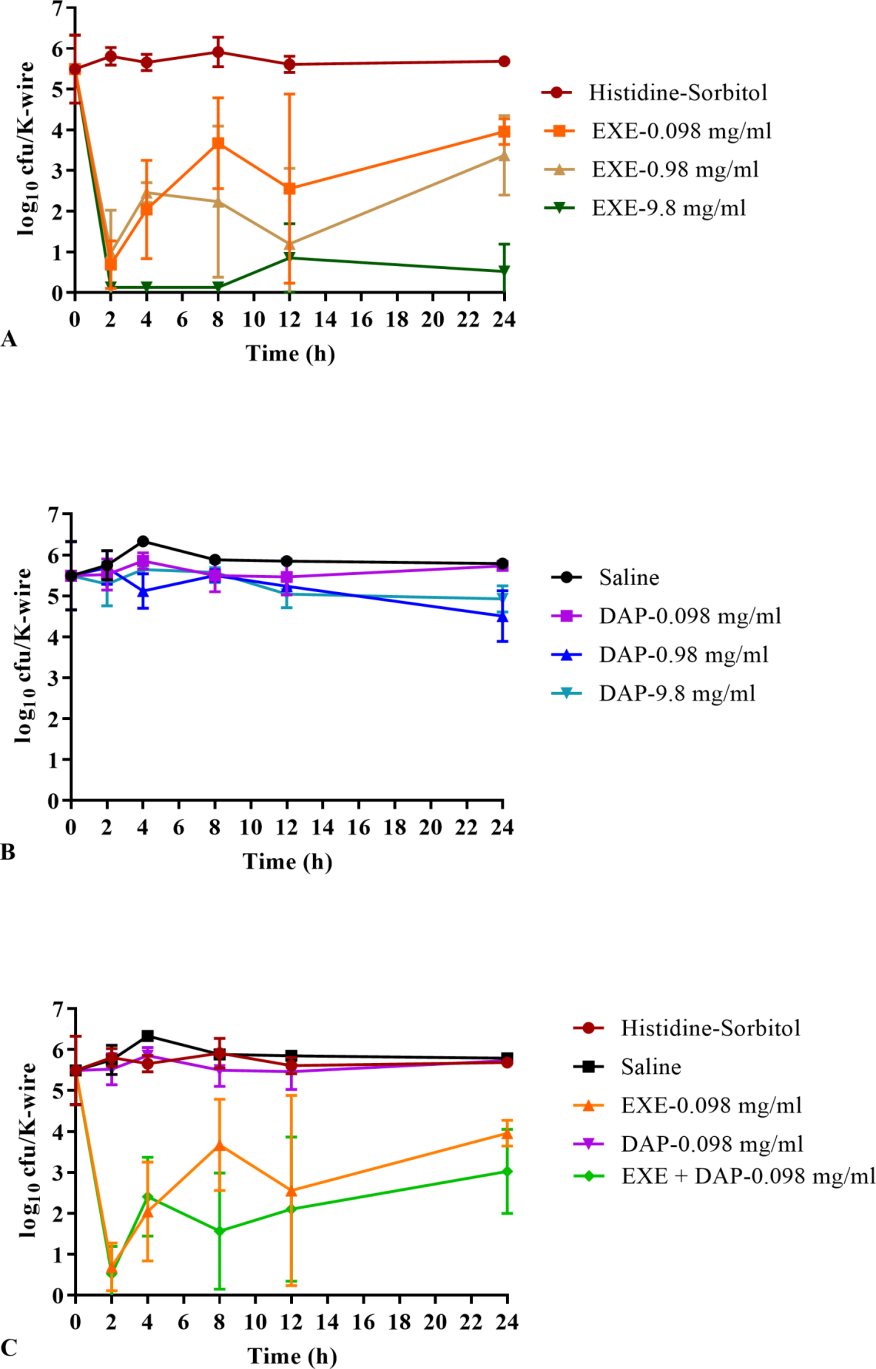
Biofilm time kill curve on orthopedic K-wires (log_10_ cfu/K-wire). (**A**) Exebacase (EXE). (**B**) Daptomycin (DAP). (**C**) Exebacase and daptomycin at 0.098 mg/ml

Biofilm time kill curves are shown in Fig. 1. The mean biofilm density was 5.49 log_10_ cfu/K-wire prior to treatment. Biofilm density of the controls remained steady throughout the time course of experiments, with biofilms in controls (exebacase and daptomycin carriers) being 5.69 and 5.79 log_10_ cfu/K-wire, respectively, at 24 h. In Fig. 1A, exebacase was more active than the control carrier solution at all concentrations and showed significant biofilm reduction at all timepoints (up to 5.78 log_10_ cfu/K-wire; P < 0.0495). Bactericidal activity (> 3 reduction of log_10_ cfu/K-wire compared to carrier control) was observed for up to 12 h for the 0.098 and 0.98 mg/ml concentrations and at 24 h for the highest concentration, with over a 5 log_10_ reduction compared to carrier control (Table 1). In Fig. 1B, daptomycin showed activity, although non-bactericidal, at all time points for 0.98 and 9.8 mg/ml and at 4 and 8 h with 0.098 mg/ml (P < 0.0495). In Fig. 1C, the combination of daptomycin (0.098 mg/ml) and exebacase (0.098 mg/ml) also showed significant biofilm reduction compared to carrier control (up to 5.29 log_10_ cfu/K-wire; P < 0.0201). The use of daptomycin in addition to exebacase did not provide a significant benefit over the bactericidal activity demonstrated for exebacase alone.

**Table 1:** Mean log_10_ cfu/K-wire reduction compared to carrier solution

Hours of treatment	Daptomycin (mg/ml)				Exebacase (mg/ml)				Daptomycin/ exebacase (mg/ml)
	0.098	0.98	9.8		0.098	0.98	9.8		0.098/0.098
2	0.23	0.06	0.46		**5.12**	**4.83**	**5.68**		**5.29**
4	0.48	1.21	0.68		**3.60**	**3.20**	**5.52**		**3.24**
8	0.39	0.3	0.31		2.24	**3.67**	**5.78**		**4.34**
12	0.38	0.61	0.81		**3.05**	**4.41**	**4.76**		**3.50**
24	0.06	1.28	0.86		1.73	2.31	**5.17**		2.66

Bolded values indicate bactericidal activity, defined as a > 3 log_10_ reduction

## Discussion

Lytic agents such as exebacase offer a potential novel mechanism to reduce the infectious burden and possibly potentiate activities of the immune system and traditional antibiotics. Previously, it was shown that exebacase reduces bacterial load when administered systemically, especially in combination with traditional antibiotics [8, 14–16]. Schuch et al. (2014) showed in vitro synergy with exebacase (referred to as CF-301) and daptomycin or vancomycin and that exebacase and daptomycin activity increased survival in a mouse *S. aureus* bacteremia model [8]. In a rat MRSA osteomyelitis model, the addition of exebacase administered systemically improved treatment outcome *versus* daptomycin alone [15].

This work provides a demonstration of bactericidal activity elicited by exebacase against MRSA biofilm on stainless steel orthopedic K-wires. These results are similar to previous work showing bactericidal activity against the same strain on titanium orthopedic cortex screws [17], suggesting that exebacase could be used for infections associated with several orthopedic implant types. We have also shown that local administration of exebacase into rabbit tibiae with implanted MRSA seeded screws reduced bacterial burden [17]. Results of this study provide support for further evaluation of local administration of exebacase as a potential treatment for orthopedic implant infections.

### Limitations

The current study was limited in the number of replicates and timepoints performed and testing of a single bacterial species and *S. aureus* strain. In addition, the study was limited to one antibiotic and one implant type. Emergence of antibiotic/lysin resistance was also not tested.

## Data Availability

Data is available from the corresponding author upon request.

## Abbreviations

cfu: Colony forming unit
DAP: Daptomycin
EXE: Exebacase
K-wire: Kirschner wire
MRSA: Methicillin-resistant *Staphylococcus aureus*
